# Genetic basis of delay discounting in frequent gamblers: examination of a priori candidates and exploration of a panel of dopamine-related loci

**DOI:** 10.1002/brb3.284

**Published:** 2014-10-16

**Authors:** Joshua C Gray, James MacKillop

**Affiliations:** 1 Department of Psychology, University of GeorgiaAthens, Georgia; 2 Boris Centre for Addictions Research, Department of Psychiatry and Behavioural Neurosciences, McMaster UniversityHamilton, Ontario, Canada

**Keywords:** Delay discounting, dopamine, endophenotype, genetics, impulsivity, pathological gambling

## Abstract

**Introduction:**

Delay discounting is a behavioral economic index of impulsivity that reflects preferences for small immediate rewards relative to larger delayed rewards. It has been consistently linked to pathological gambling and other forms of addictive behavior, and has been proposed to be a behavioral characteristic that may link genetic variation and risk of developing addictive disorders (i.e., an endophenotype). Studies to date have revealed significant associations with polymorphisms associated with dopamine neurotransmission. The current study examined associations between delay discounting and both previously linked variants and a novel panel of dopamine-related variants in a sample of frequent gamblers.

**Methods:**

Participants were 175 weekly gamblers of European ancestry who completed the Monetary Choice Questionnaire to assess delay discounting preferences and provided a DNA via saliva.

**Results:**

In a priori tests, two loci previously associated with delayed reward discounting (rs1800497 and rs4680) were not replicated, however, the long form of *DRD4* VNTR was significantly associated with lower discounting of delayed rewards. Exploratory analysis of the dopamine-related panel revealed 11 additional significant associations in genes associated with dopamine synthesis, breakdown, reuptake, and receptor function (*DRD3*, *SLC6A3*, *DDC*, *DBH*, and *SLC18A2*). An aggregate genetic risk score from the nominally significant loci accounted for 17% of the variance in discounting. Mediational analyses largely supported the presence of indirect effects between the associated loci, delay discounting, and pathological gambling severity.

**Conclusions:**

These findings do not replicate previously reported associations but identify several novel candidates and provide preliminary support for a systems biology approach to understand the genetic basis of delay discounting.

## Introduction

Although the heritability of pathological gambling (PG) is ∼50–60% (Eisen et al. 2001; Slutske et al. 2011), molecular genetic association studies have only found relatively small effects and have been inconsistent (for a review, see Lobo and Kennedy 2009). The only genome-wide association study to date was conducted on 1312 twins and found no single-nucleotide polymorphism (SNP) to reach genome-wide significance (Lind et al. 2012). These patterns are similar to findings on the genetics of other addictive disorders (Kendler et al. 2012). Given the difficulty of identifying the specific genetic contributions to PG and other addictive disorders, an endophenotype approach (Gottesman and Gould 2003) has been proposed. Unlike diagnostic phenotypes, which are polythetic and inherently heterogeneous, endophenotypes are simpler characteristics that are putatively more closely tied to a specific genetic basis within a limited number of genes. Understanding the genetic basis of these phenotypes is hoped to clarify genetic contributions to liability and to elucidate underlying mechanisms of genetic influences.

One behavioral characteristic that is considered promising is delay discounting (Mitchell 2011; Mackillop 2013), a behavioral economic measure of impulsivity that assesses a person's preferences for smaller rewards available immediately over larger delayed rewards. Delay discounting has been robustly associated with PG (e.g., MacKillop et al. 2011) and the heritability of delay discounting has been supported by evidence from studies with both animal models and human twin designs (Isles et al. 2004; Anderson and Woolverton 2005; Madden et al. 2008; Anokhin et al. 2011; Stein et al. 2012). In addition, a small number of studies have linked delay discounting with polymorphisms in genes associated with dopamine neurotransmission (Boettiger et al. 2007; Eisenberg et al. 2007; Paloyelis et al., 2010; Smith and Boettiger 2012).

The goal of the current study was to extend the understanding of the genetics of PG by examining genetic associations with delay discounting. In a sample of frequent gamblers with considerable variability in PG severity, the first goal was to examine a priori loci that have been previously associated with delay discounting, namely, *ANKK1/DRD2* TaqIA SNP rs1800497, the exon 3 variable number of tandem repeats (VNTR) polymorphism in *DRD4*, and the *COMT* SNP rs4680 (Boettiger et al. 2007; Eisenberg et al. 2007; Paloyelis et al. 2010; Smith and Boettiger 2012). The second goal of the study was an exploratory examination of associations between delay discounting and a panel of SNPs implicated in the dopamine system. To date, the existing studies have largely studied only the “usual suspects” in this area and the study sought to broaden the perspective within this system. This is also consistent with a systems biology perspective (e.g., Plomin et al. 2009; Palmer et al. 2012) in which, even for endophenotypes that are believed to be more proximal to genetic variation, it is unlikely that only a small number of polymorphisms substantially determine observed variation. Rather, genetic contributions to quantitative traits are likely to be in the form of many small effects from diverse sources of genetic variation that affect the architecture of the underlying system.

## Materials and Methods

### Participants and procedure

This study's sample comprised 178 weekly gamblers of European ancestry, who were recruited from the community via newspaper advertisements and word of mouth. Participants were screened over the phone. Inclusion criteria were: (1) weekly or greater gambling; (2) 18–65 years old. Exclusionary criteria were: (1) currently living with someone who already completed the study; (2) computer illiteracy; (3) psychotic symptoms. Three participants were excluded due to unsuccessful genotyping (see below), leaving a final sample of 175.

After completing the informed consent, participants completed a diagnostic interview for PG, a variety of self-report questionnaires, including a delay discounting task, and submitted a DNA sample. Following participation, participants rolled a six-sided die to determine if they would receive one randomly selected outcome from their choices on the delay discounting task (Kirby et al. 1999), provided in cash either immediately or after the delay. Additionally, participants were compensated $30 for their participation. All procedures were approved by the University of Georgia Institutional Review Board.

### Measures

#### Demographics

Comprehensive demographics were assessed including sex, age, race, gender, income, education, and other descriptive variables.

#### Pathological gambling

The Structured Clinical Interview for Pathological Gambling (SCI-PG) (Grant et al. 2004) is a semistructured interview that was used to assess participants’ current and heaviest gambling periods. The SCI-PG is based on the 10 *DSM-IV* symptoms of pathological gambling and was administered by a trained MS-level clinician. Pathological gambling symptoms were treated as a continuous variable given evidence indicating the dimensionality of PG (Strong and Kahler 2007; Goodie et al. 2013).

#### Delayed reward discounting

Participants were administered the Monetary Choice Questionnaire (MCQ) (Kirby et al. 1999), a widely used measure that consists of 27 randomized choices between smaller immediate rewards and larger delayed rewards. The rewards ranged from $11 to $85 and the larger delayed rewards were available at varying intervals of delay from 1 week to 186 days.

#### Genotyping

**Figure 1:**
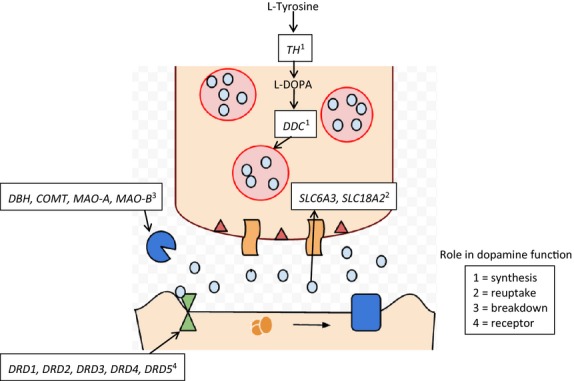
Genes selected based on roles in functional aspects of dopamine neurotransmission.

For DNA collection, a saliva sample was obtained from each participant using Oragene OG-5000 collection kits (DNA Genotek, Ottawa, ON, Canada). Sufficient DNA for the candidate polymorphisms was extracted from 100% of the saliva samples. Genotyping comprised 236 dopamine-related SNPs, the two a priori SNPs and DRD4 VNTR. The exploratory SNPs were loci in genes responsible for diverse aspects of dopamine neurotransmission (Fig.1), including the five receptor genes and also the genes responsible for dopamine synthesis, breakdown, and reuptake. Specifically, the loci were tag SNPs that came from the dopamine section of the addictions array by National Institute on Alcohol Abuse and Alcoholism (Hodgkinson et al. 2008) and were supplemented by loci from other recent studies exploring variation based on dopamine-related genes (Munafò et al. 2004; Berrettini and Lerman 2005; Kreek et al. 2005; Gelernter et al. 2006; Nackley et al. 2006; Yu et al. 2006; Zhang et al. 2006; Dick et al. 2007; Ho and Tyndale 2007; Bergen et al. 2009). The SNP genotyping was conducted using a custom panel on an Illumina BeadXpress (Illumina, San Diego, CA). The *DRD4* VNTR genotyping was conducted using PCR with the primers forward 5′-CGA CTA CGT GGT CTA CTC G-3′ and reverse 5′-/56-FAM/AGG ACC CTC ATG GCC TTG-3′. Determination of the allele length was performed by analyses on an automated capillary sequencer (AB13730xl, Applied Biosystems, Burlington, ON, Canada) and genotype was then called using GeneMapper 4.0 (Applied Biosystems).

### Statistical analysis

An estimate of a participant's level of impulsivity (i.e., *k*) can be made from the participant's pattern of choices across the 27 MCQ questions (Kirby et al. 1999). The *k* value in this case reflects the inferred hyperbolic discounting function that is most consistent with the participants’ choices. For example, a person with a discount rate of 0.10 would be indifferent between “$33 today” and “$80 in 14 days,” so if they chose the smaller immediate reward, then they would have a discounting rate greater than 0.10. In a question where the immediate reward is less and the delayed reward is larger and sooner (e.g., “$31 today” or “$85 in 7 days”), a discounting rate of 0.25 would demonstrate indifference between those two rewards. If the participant chose the delayed reward here, then they would have a discounting rate less than 0.25. From these two trials, it could be inferred that the participant has a discount rate between 0.10 and 0.25 and the geometric mean of the two is taken to avoid underweighting the smaller value (in this example, *k = *0.16). In the event that more than two *k* values are equally consistent, the geometric mean of the values was computed. The measure has three sets of nine items grouped into three delayed reward sizes: small ($25 to $35), medium ($50 to $60), and large ($75 to $85); items from all three sizes are mixed together. Separate *k* values were calculated for small, medium, and large delayed rewards. However, because they were very highly correlated (large–medium, *r *=* *0.92; large–small, *r *=* *0.84; medium–small, *r *=0* *.89; *P* < 0.001), to reduce the number of statistical tests, a single average *k* value was used. Finally, to improve its positively skewed distribution, *k* was log10 transformed.

The software PLINK (RRID: nlx_154200) was used to examine genotype–phenotype associations (Purcell et al. 2007). For a priori loci, the analyses were based on empirical precedents. Specifically, for *ANKK1/DRD2* TaqIA (rs1800497), possession of the T allele was predicted to be associated with significantly greater delay discounting using a dominance model (Eisenberg et al. 2007). This effect was further predicted to be moderated by possession of the long form of the *DRD4* VNTR gene (Eisenberg et al. 2007), with *DRD4* VNTR status being dichotomized into presence (7+ allele carriers) and absence (<7 allele homozygotes), again in a dominance model. However, given the exploratory nature of this study, we also investigated the significance of rs1800497 and *DRD4* VNTR using an additive model. *DRD4* VNTR and rs1800497 were centered about the mean prior to conducting moderation analysis. Then *DRD4* VNTR and rs1800497 were entered into the second step of a hierarchical linear regression model (income was entered into the first step), followed by the interaction effects of *DRD4* VNTR and rs1800497. With regard to COMT val158met (rs4680), we did not make a specific allelic prediction based on the previously conflicting findings (Boettiger et al. 2007; Paloyelis et al. 2010; Smith and Boettiger 2012).

For the exploratory analyses, the number of minor alleles (i.e., 0, 1, or 2) was examined in relation to the phenotype using an additive model for maximum resolution. Regression analyses for testing SNP and dichotomized VNTR with delay discounting were conducted using standard significance values (*P *≤* *0.05). Following the individual a priori and exploratory SNP analyses, we examined all significant SNPs summed into an AGRS, in relation to delay discounting. Aggregate genetic risk scores were calculated using the following formula: AGRS = (sum of risk allele scores/number of nonmissing genotypes × 2) × (2 × total number of SNPs in the AGRS) (Cornelis et al. 2009). This simple count method of calculating the AGRS assumes an additive genetic model where equivalent effects of each polymorphism and pathological gambling are expected. This model does not allow for epistatic effects. For each significant SNP, participants were given a score (i.e., 0, 1, or 2) denoting the number of risk alleles they possessed.

Finally, to examine whether delay discounting was indirectly responsible for the relationship between genetic variation and PG severity, we conducted mediational analyses for all SNPs significantly associated to delay discounting. In the absence of a significant association between the independent variable and dependent variable, the significance of the indirect effect was still tested because the direct relationship may not be present due to low power or suppression effects (Mackinnon and Fairchild 2009). Indirect effects were assessed using bootstrapped confidence intervals (Preacher and Hayes 2008; *n *=* *1000, 95% confidence intervals [CIs]). This procedure overcomes the potential violation of the assumption of multivariate normality inherent in tests of indirect effects that use product terms and it allows for statistical control of covariates (i.e., income). The absence of zero in the confidence interval indicates significant indirect effects. The mediational relationship between the AGRS, delay discounting, and pathological gambling was also examined.

## Results

### Preliminary analyses

Three participants were excluded for missing >15% genotypes (final *N *=* *175). Participant characteristics were: 86.3% male; age *M *=* *34.7, SD* = *13.1; years of education *M *=* *13.5, SD = 2.5; recreational gamblers [no symptoms] *n *=* *48 (27%); problem gamblers [1–4 symptoms] *n *=* *82 (47%); pathological gamblers [5+ symptoms] *n *=* *45 (26%). With regard to genotyping, of an initial panel of 236 dopamine-related loci genotyped, SNPs with excessive numbers of nonviable samples (>20%) and insufficient variability (minor allele frequency [MAF] <10%) were excluded from further consideration, leaving 153 SNPs. Hardy–Weinberg equilibrium (HWE) was tested to identify abnormal frequencies, however, given the selected nature of the sample (i.e., frequent gamblers), SNPs were not excluded prior to analyses for abnormal frequencies (Sham 1998), although the a priori loci were all in HWE. Detailed characteristics (including HWE) of all SNPs used in analyses are reported in supplementary materials. Correlations among phenotypes are depicted in Table 1. Income was significantly associated with both phenotypes of interest (i.e., PG severity and log*k*) and therefore it was included as a covariate in all following analyses. The significant relationship between PG severity and log*k* remained significant when controlling for income (*r *=* *0.31, *P *<* *0.01).

**Table 1:** Associations among delay discounting, severity of pathological gambling (PG), and income (*N* = 175).

Variable	*M* (SD)/Median	1	2
1 log10*k*	−1.43 (0.69)	–	–
2 PG severity	2.8 (2.8)	0.34**	–
3 Income	$15,000–$29,999	−0.25**	−0.19*

*k *= behavioral economic index of impulsivity; severity reflects number of DSM-IV symptoms;

* *P *<* *0.05,

** *P *<* *0.01.

### A priori loci

Associations between delay discounting and the a priori loci are presented in Table 2. Possession of the long form of *DRD4* VNTR was found to be significantly associated with lower log*k* (i.e., less discounting of delayed rewards) (*P *<* *0.05). No statistically significant associations were found between the a priori loci *ANKK1/DRD2* (rs1800497) and *COMT* (rs4680) and log*k*. Analyses were conducted examining if there was a moderating effect of possession of the long form (7R) of *DRD4* VNTR on the association between possession of the minor allele (T) of rs1800497 and log*k*. When including income in the first step of a linear regression model, both genes in the second step, and the interaction effects in the third step, no significant moderating effects were found (*P *=* *0.43; see Table 2). Analyses of the direct relationships between *DRD4* VNTR and rs1800497 and log*k* using an additive model yielded analogous results: *DRD4* VNTR was significantly associated (*P *=* *0.04), while rs1800497 was nonsignificantly associated (*P *=* *62).

**Table 2:** Candidate gene associations with delay discounting (DD), pathological gambling severity (PG), and mediational analyses.

Genetic variables					DD (log*k*)			PG severity			Indirect effect			
Chromosome	Gene	Polymorphism	Mi/Ma	MAF	*B*	*t*	*P*	*B*	*t*	*P*	*Estimate*	SE	Lower CI	Upper CI
A priori Loci														
11	DRD4^1^	VNTR^2^	7R+/<7R	0.14	−0.29	−2.00	**0.05**	−0.19	−0.31	0.75	−0.38	0.27	−1.00	0.08
11	ANKK1	rs1800497^2^	T/C	0.20	0.08	0.76	0.45	0.80	1.8	0.07	–	–	–	–
22	COMT	rs4680	G/A	0.42	−0.02	−0.23	0.82	−0.30	−1.00	0.32	–	–	–	–
	DRD4xANKK1				0.24	0.79	0.43	0.29	0.23	0.82	–	–	–	–
Exploratory panel														
3	DRD3^1^	rs3773678	T/C	0.17	−0.19	−2.20	**0.03**	−0.02	−0.05	0.96	−0.25	0.16	−0.63	0.01
3	DRD3^1^	rs7638876	G/A	0.35	−0.14	−2.10	**0.04**	−0.04	−0.15	0.88	−0.19^4^	0.10	−0.42	−0.03
5	SLC6A3^1^	rs464049	G/A	0.45	−0.17	−2.38	**0.02**	−0.61	−2.07	**0.04**	−0.20^4^	0.09	−0.40	−0.04
5	SLC6A3^1^	rs3756450	G/A	0.12	−0.29	−2.68	**0.01**	−0.43	−0.94	0.35	−0.37^4^	0.17	−0.77	−0.10
5	SLC6A3^1^	rs12652860^3^	A/C	0.27	0.20	2.42	**0.02**	0.31	0.90	0.37	−0.25^4^	0.11	−0.50	−0.06
7	DDC^1^	rs10249982	C/T	0.22	−0.18	−2.08	**0.04**	−0.17	−0.48	0.63	−0.23	0.13	−0.53	0.00
7	DDC^1^	rs10244632^3^	T/C	0.25	−0.20	−2.64	**0.01**	−0.22	−0.67	0.50	−0.26^4^	0.12	−0.54	−0.07
7	DDC^1^	rs1466163	A/G	0.11	−0.23	−1.99	**0.05**	−0.85	−1.80	0.07	−0.28	0.18	−0.72	0.01
7	DDC^1^	rs10499696	C/T	0.11	−0.28	−2.48	**0.01**	−0.81	−1.71	0.09	−0.34^4^	0.19	−0.80	−0.05
9	DBH^1^	rs2519154	C/T	0.45	−0.16	−2.34	**0.02**	0.03	0.10	0.92	−0.22^4^	0.11	−0.48	−0.04
10	SLC18A2^1^	rs363338	G/A	0.30	−0.21	−2.70	**0.01**	−0.21	−0.65	0.52	−0.27^4^	0.13	−0.56	−0.07
Aggregate Genetic Risk Score (AGRS)					0.10	6.18	**<0.001**	0.14	1.92	0.06	0.12^4^	0.04	0.06	0.21

Significant associations are highlighted in boldface. Alleles are coded higher for larger numbers of minor alleles; income was included as an covariate; indirect effects were based on 1000 bootstrap samples; and 95% confidence intervals (CIs) were used to test significance.

1 Included in AGRS.

2 Dominance model based on previous studies, all other analyses used additive models.

3 *n *=* *174.

4 Indirect effect is significantly different from 0.

### Exploratory panel

Detailed results of individual associations between exploratory loci and delay discounting can be found in Table 2 and the supplementary materials. Eleven novel SNPs from genes *DRD3*, *SLC6A3*, *DDC*, *DBH*, and *SLC18A2* were significantly associated with log*k* at *P *<* *0.05. An AGRS including the 11 significant SNPs and *DRD4* VNTR (maximum possible AGRS = 24) was calculated. Results yielded a significant association between this AGRS and log*k* after controlling for income (*R*^2^ = 0.17, *P *<* *0.001).

### Mediational analyses

Mediational analyses are presented in Table 2. Notably, one individual SNP that was significantly associated with log*k*, rs464049, was also significantly associated with PG directly, and this relationship was significantly mediated by log*k*. Furthermore, in the mediational model the relationship between rs464049 and PG severity was reduced to nonsignificance (*P *=* *0.16), suggesting full mediation by log*k*. In all other cases where there were nonsignificant relationships between the SNPs and PG, and testing of indirect effects was still conducted to determine whether delay discounting was the variable through which the SNPs contributed to variance in PG severity. Analyses revealed significant indirect effects between 7 of the 10 remaining loci and PG by log*k*. Notably, there was no significant mediation of *DRD4* VNTR and PG severity by log*k* (regardless of whether an additive or dominance model was examined). The AGRS was associated with PG at a trend level and this relationship was significantly mediated by log*k*.

## Discussion

This study sought to extend the literature on the genetic basis of delay discounting using several strategies. In a novel sample of frequent gamblers we examined three a priori candidate polymorphisms (*DRD4* VNTR, *ANKK1/DRD2* [rs1800497], *COMT* [rs4680]), explored a panel of loci associated with dopamine neurotransmission, and integrated the observed associations using an AGRS strategy. In addition, we conducted mediational analyses to evaluate the extent to which the association between genetic variation and delay discounting was indirectly responsible for the relationship between the variants and severity of pathological gambling. Evidence of this relationship would support the hypothesis that impulsive delay discounting serves as an intermediate mechanism between genetic variation and the development of pathological gambling.

For the three a priori polymorphisms, we identified a significant association between the long form of *DRD4* VNTR and less impulsive discounting. This is interesting but somewhat surprising, as this is the first study to identify a direct association with this locus and is inconsistent with two previous studies. In one, *DRD4* VNTR was not significantly associated with discounting but exhibited an epistatic interaction with rs1800497 such that individuals who were carriers of both the T allele of rs1800497 and the long form of *DRD4* VNTR exhibited substantially more impulsive discounting (Eisenberg et al. 2007). It was also notable that the current study did not replicate the association between rs1800497 and delay discounting in that study. In a second study, *DRD4* VNTR was simply not associated with level of discounting (Garcia et al. 2010). It may be that the discrepancies are attributable to several sample differences. The samples from these two past studies comprised comparable numbers of subjects (195 and 181, respectively), however, the samples were healthy, young college students, whereas this study comprised older, low SES, weekly gamblers recruited from the community.

With regard to rs4680, no significant association was present, which is also somewhat surprising as that locus has been significantly associated with an individual's level of discounting in three previous studies (Boettiger et al. 2007; Paloyelis et al. 2010; Smith and Boettiger 2012). However, those studies are themselves inconsistent, with two finding G/G as the risk genotype and one finding A/A as the risk genotype. These conflicting findings have been reconciled from a developmental perspective (Smith and Boettiger 2012), with G/G genotype appearing to be the risk genotype among adults, however that does not address the absence of a significant association in the current study. Again, notable methodological differences are present between the current study and the previous ones, which used different tasks and smaller samples (ranging between 19 and 72 subjects) including healthy individuals, individuals with previous alcohol use disorders, and adolescents with attention deficit hyperactivity disorder. Taken together, the results focusing on previously reported associations did not replicate those findings, suggesting either more nuanced or unstable relationships.

For the exploratory strategy, a systems biology approach was applied in which a panel of tag SNPs in genes responsible for diverse aspects of dopamine neurotransmission was examined. The goal here was to broaden the scope of genetic variants considered and also to integrate the observed associations into a cumulative model. Eleven novel markers significantly accounting for variance in delay discounting were identified and were located in genes responsible for dopamine synthesis, degradation, reuptake, and receptor functionality (*DRD3*, *SLC6A3*, *DDC*, *DBH*, and *SLC118A2*). Interestingly, in most of the cases, possession of the minor allele was significantly associated with less impulsive discounting, not more. For most of these loci, the molecular functionality and behavioral relevance have not been fully characterized, but there is some existing literature that informs these significant associations.

The *DRD3* gene encodes the dopamine D_3_ receptor, which is responsible for the inhibition of intracellular cyclic AMP and is expressed primarily in regions of the limbic system (Pierce and Kumaresan 2006). There is evidence that D_3_ receptor-deficient mice demonstrate increased hyperactivity and sensitivity to reward that predisposes them to impulsive drug taking behavior (Le Foll et al. 2005). Interestingly, rs3773678 in *DRD3* has been associated with nicotine dependence, although not consistently (Huang et al. 2008; Wei et al. 2012), and has been associated with performance on the Continuous Performance Task (CPT), another behavioral measure of impulsivity (Kollins et al. 2008). Also in *DRD3*, rs7638876 was found in one study to be in a haplotype that was significantly associated with smoking phenotypes (Huang et al. 2008). Among the *SLC6A3* SNPs, there is evidence that rs3756450 may be the primary active SNP, as one study utilizing electrophoretic mobility shift assays identified allele-specific binding in rs3756450 alone (Talkowski et al. 2008; Bamne et al. 2010). Notably, rs464049 has been found to be significantly associated with smoking-related behavior (Caporaso et al. 2009). In addition, rs12652860 has been significantly associated with nicotine dependence (Bergen et al. 2009). In general, these SNPs have received relatively little attention to date yet the existing literature reveals links to other aspects of impulsivity and to addictive behavior.

When the variants were aggregated, the AGRS was significantly associated with delay discounting above the effect sizes attributable to any individual SNPs and accounted for a meaningful proportion of variance. This is highly consistent with a systems approach in which possession of any single risk variant may not translate directly to notably elevated expression of a phenotype, but possession of larger numbers of small-effect-size risk variants additively give rise to meaningful elevations of the characteristic (Plomin et al. 2009; McGeary et al. 2012). However, it is important to note that this was a a post hoc descriptive analysis, not an a priori predictive analysis. Aggregating individually significant loci would be assumed to create a statistically significant model; what is of greatest interest is the amount of variation captured by the aggregated loci.

The mechanistic analyses revealed significant mediational roles between the genetic variables, delay discounting, and gambling severity to a large extent. In particular, full mediation was present between rs464049 and PG severity by impulsive discounting. Furthermore, in 7 of the 11 other loci, significant indirect effects were present, supporting delay discounting as a pathway through which the loci incrementally contributed variance to PG severity. The trend-level association between the AGRS and PG was also significantly mediated by delayed discounting. This finding provides preliminary evidence that individuals with multiple dopamine-related risk alleles for delay discounting also have more PG symptoms and this relationship is accounted for by the relationship of these dopamine SNPs and delay discounting. Furthermore, because delay discounting is a full mediator of the relationship, this finding renders it unlikely that AGRS is having a pleiotropic effect on PG (i.e., there is no independent AGRS–PG association). Together, these findings provide initial support for delay discounting as an intermediate risk variable for genetic influences on PG.

It is important to note that the study had several limitations that bear consideration. First, for the exploratory arm of the study, a relatively large number of novel loci were examined in a relatively small sample of participants, creating inflation of type I error rate. As such, several significant associations would be expected to emerge by chance alone and the identified associations would not survive stringent type I error correction (e.g., Bonferroni). As this aspect of the study was intentionally exploratory, error correction was not implemented, but caution should be applied in interpreting these findings and replication of these associations will be essential. Of note, this issue is mitigated slightly by the fact that the number of significant associations substantially surpassed the number expected by chance and the mediational analyses supported mechanistic relationships, which would not be expected for randomly occurring associations. However, another limitation pertains to the mediational analyses. Although those findings generally suggested discounting in an intermediate mechanistic role, it is important to note that the cross-sectional study design limits the strength of causal inference. Definitive evidence that genetic factors give rise to elevated delay discounting that, in turn, gives rise to PG would necessarily require a longitudinal design and could not be addressed in this study. Finally, although we minimized the risk of population stratification by only examining individuals of European ancestry, we were not able to examine SNPs associa-ted with impulsive discounting that may be common in non-Europeans but rare among individuals of European ancestry. Despite its limitations, this study extends the literature on the genetic basis of delay discounting, both in general and as a risk mechanism for addictive behavior. Although previous associations were not replicated, the study identified a number of new loci that appear to be associated with delay discounting and provides proof of concept for an aggregate genetic risk score approach.

The existing literature on the molecular genetics of delay discounting is small in terms of studies and, within that paucity, the studies have been small in terms of sample size. Going forward, there is a need for studies with considerably larger sample sizes to replicate the current associations and clarify the mixed findings in the literature. Furthermore, future research should explore the genetic basis of other relevant possible endophenotypes, such as risk taking (e.g., probability discounting). Finally, this study only considered the dopaminergic system and therefore future studies should seek to explore the role of other upstream systems implicated in the reward pathway such as GABA and glutamate, which are known to innervate dopamine neurons in central regions of the mesolimbic system, the ventral tegmental area and the nucleus accumbens (Nestler 2005).

## Supporting Information

**Table S1.** Single-nucleotide polymorphism (SNP) characteristics and associations with delay discounting (DD).
