# Supplementary material for: Genetic basis of delay discounting in frequent gamblers: examination of a priori candidates and exploration of a panel of dopamine-related loci
Source: Brain Behav. 2014 Oct 16;4(6):812–21. doi: 10.1002/brb3.284 (PMC4212112; doi:10.1002/brb3.284)
Supplement: Supplementary file 1 — Table S1. Single-nucleotide polymorphism (SNP) characteristics and associations with delay discounting (DD). [file brb30004-0812-sd1.docx]

**SUPPLEMENTARY MATERIALS**

Supplementary Table 1

*Single-nucleotide polymorphism (SNP) characteristics and associations with delay discounting (DD).*

| Chr | Gene | SNP | BP Position | Participants | MAF | Min | Maj | HWE | DD (log*k)* | | |
| --- | --- | --- | --- | --- | --- | --- | --- | --- | --- | --- | --- |
|  |  |  |  |  |  |  |  |  | *β* | *t* | *p* |

| 3 | DRD3 | rs2134655 | 113858201 | 175 | .27 | A | G | 0.08 | .01 | .73 | .47 |
| --- | --- | --- | --- | --- | --- | --- | --- | --- | --- | --- | --- |
| 3 | DRD3 | rs963468 | 113862887 | 175 | .39 | A | G | 1.00 | .01 | 1.08 | .28 |
| 3 | DRD3 | rs3773678 | 113870078 | 175 | .17 | T | C | 0.01 | -.01 | -.93 | .35 |
| 3 | DRD3 | rs167770 | 113879562 | 174 | .29 | G | A | 0.03 | -.01 | -1.38 | .17 |
| 3 | DRD3 | rs324029 | 113881623 | 174 | .29 | T | C | 0.03 | -.01 | -1.39 | .17 |
| 3 | DRD3 | rs10934256 | 113885652 | 174 | .2 | T | G | 0.64 | -.01 | -1.34 | .18 |
| 3 | DRD3 | rs7633291 | 113887068 | 175 | .2 | C | A | 0.64 | -.01 | -1.29 | .20 |
| 3 | DRD3 | rs324022 | 113887298 | 175 | .29 | T | C | 0.02 | -.01 | -1.55 | .12 |
| 3 | DRD3 | rs7638876 | 113894300 | 175 | .35 | G | A | 0.03 | -.01 | -1.54 | .13 |
| 3 | DRD3 | rs9825563 | 113900220 | 174 | .33 | C | T | 0.23 | -.01 | -.82 | .41 |
| 4 | DRD5 | rs7655090 | 9765875 | 175 | .28 | G | A | 0.35 | .00 | -.25 | .80 |
| 4 | DRD5 | rs10939515 | 9773296 | 175 | .14 | T | C | 0.05 | -.01 | -.80 | .43 |
| 4 | DRD5 | rs2867383 | 9787935 | 175 | .3 | A | G | 1.00 | .00 | -.08 | .94 |
| 4 | DRD5 | rs13106539 | 9797703 | 175 | .34 | C | T | 0.31 | .01 | .97 | .33 |
| 5 | SLC6A3 | rs6347 | 1411412 | 175 | .26 | G | A | 0.17 | .00 | .18 | .86 |
| 5 | SLC6A3 | rs27048 | 1412645 | 172 | .45 | A | G | 0.76 | .00 | -.20 | .84 |
| 5 | SLC6A3 | rs37022 | 1415629 | 175 | .19 | T | A | 0.01 | .00 | .08 | .93 |
| 5 | SLC6A3 | rs464049 | 1423905 | 175 | .45 | G | A | 1.00 | -.01 | -1.21 | .23 |
| 5 | SLC6A3 | rs403636 | 1438354 | 175 | .13 | T | G | 0.74 | .00 | .05 | .96 |
| 5 | SLC6A3 | rs2652511 | 1446389 | 168 | .41 | A | G | 0.42 | .00 | -.02 | .99 |
| 5 | SLC6A3 | rs2652510 | 1447860 | 174 | .39 | C | T | 0.75 | .00 | .49 | .63 |
| 5 | SLC6A3 | rs3756450 | 1448148 | 175 | .12 | G | A | 0.72 | -.02 | -1.34 | .18 |
| 5 | SLC6A3 | rs12652860 | 1453772 | 174 | .27 | A | C | 1.00 | .01 | 1.57 | .12 |
| 5 | DRD1 | rs686 | 174868700 | 175 | .41 | C | T | 0.64 | -.01 | -1.80 | .07 |
| 5 | DRD1 | rs5326 | 174870196 | 175 | .15 | A | G | 0.38 | .00 | .37 | .71 |
| 7 | DDC | rs4947510 | 50525420 | 174 | .28 | A | G | 0.26 | .00 | -.17 | .87 |
| 7 | DDC | rs4947535 | 50531681 | 175 | .3 | T | A | 0.59 | .00 | .13 | .89 |
| 7 | DDC | rs732215 | 50544063 | 148 | .43 | C | A | 0.62 | .00 | -.37 | .71 |
| 7 | DDC | rs4490786 | 50544314 | 175 | .19 | A | G | 1.00 | -.01 | -.87 | .38 |
| 7 | DDC | rs2122822 | 50552152 | 175 | .4 | G | C | 0.21 | -.01 | -.62 | .54 |
| 7 | DDC | rs880028 | 50570136 | 175 | .19 | G | A | 1.00 | -.01 | -.86 | .39 |
| 7 | DDC | rs10249982 | 50591390 | 175 | .22 | C | T | 0.82 | -.02 | -1.80 | .07 |
| 7 | DDC | rs10244632 | 50598703 | 174 | .25 | T | C | 0.16 | -.02 | -1.97 | .05 |
| 7 | DDC | rs1466163 | 50607206 | 175 | .11 | A | G | 1.00 | -.02 | -1.46 | .15 |
| 7 | DDC | rs7786398 | 50612906 | 175 | .45 | G | A | 0.88 | -.01 | -1.18 | .24 |
| 7 | DDC | rs2329341 | 50620275 | 175 | .31 | C | A | 0.73 | .01 | .71 | .48 |
| 7 | DDC | rs10499696 | 50621588 | 175 | .11 | C | T | 1.00 | -.02 | -1.77 | .08 |
| 7 | DDC | rs3829897 | 50629764 | 174 | .39 | A | C | 0.43 | .00 | .45 | .66 |
| 7 | DDC | rs7804365 | 50637148 | 175 | .5 | C | A | 1.00 | .00 | .48 | .63 |
| 7 | DDC | rs12669770 | 50656334 | 175 | .34 | T | C | 1.00 | .01 | .79 | .43 |
| 9 | DBH | rs1076153 | 136498143 | 175 | .19 | T | G | 0.09 | -.01 | -1.41 | .16 |
| 9 | DBH | rs1076150 | 136498761 | 167 | .5 | C | T | 0.76 | .01 | 1.54 | .13 |
| 9 | DBH | rs1611114 | 136500203 | 171 | .28 | A | G | 0.00 | -.01 | -.96 | .34 |
| 9 | DBH | rs2797849 | 136501941 | 174 | .35 | G | C | 0.62 | .00 | .57 | .57 |
| 9 | DBH | rs3025388 | 136503256 | 175 | .17 | G | A | 1.00 | .01 | .60 | .55 |
| 9 | DBH | rs2007153 | 136503819 | 174 | .36 | A | G | 0.10 | .01 | .75 | .45 |
| 9 | DBH | rs2873804 | 136505644 | 172 | .45 | T | C | 0.22 | -.01 | -.81 | .42 |
| 9 | DBH | rs1611124 | 136509275 | 174 | .1 | A | C | 1.04 x 10^-25^ | -.01 | -.68 | .49 |
| 9 | DBH | rs1541332 | 136511516 | 175 | .46 | T | C | 0.45 | .00 | .18 | .86 |
| 9 | DBH | rs2519154 | 136512275 | 175 | .45 | C | T | 0.55 | -.01 | -1.28 | .20 |
| 9 | DBH | rs77905 | 136518097 | 175 | .45 | T | C | 0.65 | .01 | 1.30 | .19 |
| 9 | DBH | rs2073833 | 136520282 | 172 | .46 | G | C | 0.76 | -.01 | -1.16 | .25 |
| 9 | DBH | rs1611131 | 136522187 | 175 | .3 | G | A | 0.37 | -.01 | -.58 | .56 |
| 9 | DBH | rs2073837 | 136522928 | 175 | .31 | T | C | 0.05 | -.01 | -1.12 | .26 |
| 9 | DBH | rs129882 | 136523669 | 136 | .18 | A | G | 0.57 | .00 | -.05 | .96 |
| 9 | DBH | rs129915 | 136524918 | 175 | .29 | G | A | 0.28 | -.01 | -.60 | .55 |
| 10 | SLC18A2 | rs363332 | 119002667 | 175 | .21 | A | G | 1.00 | -.02 | -1.50 | .13 |
| 10 | SLC18A2 | rs363334 | 119004995 | 175 | .22 | G | C | 1.00 | -.02 | -1.67 | .10 |
| 10 | SLC18A2 | rs363338 | 119009389 | 175 | .3 | G | A | 1.00 | -.02 | -2.48 | .01 |
| 10 | SLC18A2 | rs4752045 | 119019690 | 174 | .42 | C | G | 0.53 | .00 | .18 | .86 |
| 10 | SLC18A2 | rs2015586 | 119021737 | 174 | .26 | C | T | 4.63 x 10^-14^ | .00 | -.29 | .78 |
| 10 | SLC18A2 | rs363230 | 119029515 | 175 | .46 | A | G | 0.45 | .00 | -.50 | .62 |
| 10 | SLC18A2 | rs2244249 | 119032275 | 152 | .13 | T | C | 0.48 | -.01 | -.77 | .44 |
| 10 | SLC18A2 | rs363276 | 119033809 | 175 | .14 | A | G | 0.76 | .00 | .04 | .97 |
| 11 | DRD4 | rs7932167 | 620599 | 174 | .23 | C | A | 0.39 | .01 | .89 | .37 |
| 11 | DRD4 | rs3758653 | 636399 | 175 | .17 | G | A | 1.00 | .02 | 1.42 | .16 |
| 11 | TH | rs2070762 | 2186335 | 174 | .5 | A | G | 0.88 | -.01 | -.78 | .43 |
| 11 | TH | rs6356 | 2190951 | 175 | .33 | A | G | 0.87 | .00 | -.30 | .76 |
| 11 | NCAM1 | rs2011505 | 112988236 | 174 | .43 | C | T | 0.09 | -.01 | -1.24 | .22 |
| 11 | NCAM1 | rs1245119 | 113001661 | 175 | .39 | G | C | 0.04 | .00 | .10 | .92 |
| 11 | NCAM1 | rs2043602 | 113043726 | 175 | .39 | C | T | 0.63 | .00 | -.41 | .68 |
| 11 | NCAM1 | rs2117912 | 113060469 | 173 | .22 | C | T | 0.50 | .00 | -.47 | .64 |
| 11 | NCAM1 | rs1821693 | 113077541 | 175 | .41 | C | T | 0.64 | -.01 | -1.06 | .29 |
| 11 | NCAM1 | rs686934 | 113085448 | 175 | .41 | G | A | 0.64 | -.01 | -1.10 | .27 |
| 11 | NCAM1 | rs584427 | 113103996 | 175 | .5 | T | G | 0.45 | .00 | .24 | .81 |
| 11 | NCAM1 | rs646558 | 113105907 | 174 | .22 | T | G | 0.18 | .01 | 1.30 | .20 |
| 11 | NCAM1 | rs586903 | 113110946 | 175 | .13 | C | A | 0.32 | -.01 | -.53 | .60 |
| 11 | NCAM1 | rs2303377 | 113111501 | 175 | .44 | G | A | 0.44 | .00 | -.22 | .82 |
| 11 | NCAM1 | rs605843 | 113125234 | 174 | .29 | C | T | 0.20 | -.01 | -1.40 | .16 |
| 11 | NCAM1 | rs2288158 | 113133676 | 175 | .16 | C | A | 0.58 | -.02 | -1.75 | .08 |
| 11 | NCAM1 | rs598026 | 113139250 | 175 | .15 | G | A | 0.77 | -.01 | -.84 | .40 |
| 11 | NCAM1 | rs2156485 | 113143557 | 174 | .25 | T | C | 0.11 | .00 | .27 | .79 |
| 11 | NCAM1 | rs593217 | 113153489 | 175 | .45 | C | T | 0.65 | .01 | 1.62 | .11 |
| 11 | NCAM1 | rs688011 | 113154170 | 175 | .31 | A | G | 0.48 | -.01 | -.89 | .38 |
| 11 | NCAM1 | rs7103866 | 113164768 | 170 | .28 | T | C | 0.71 | .00 | .35 | .73 |
| 11 | TTC12 | rs1893699 | 113192524 | 172 | .39 | C | A | 1.00 | .01 | 1.52 | .13 |
| 11 | TTC12 | rs723077 | 113194168 | 175 | .45 | C | A | 1.00 | -.01 | -.84 | .40 |
| 11 | TTC12 | rs2303380 | 113200709 | 175 | .36 | G | A | 0.51 | .02 | 1.88 | .06 |
| 11 | TTC12 | rs948176 | 113204481 | 175 | .19 | G | A | 0.63 | -.01 | -.93 | .35 |
| 11 | TTC12 | rs2288159 | 113211329 | 169 | .2 | A | C | 0.81 | -.01 | -1.12 | .26 |
| 11 | TTC12 | rs719804 | 113234775 | 174 | .23 | G | A | 0.40 | .01 | 1.11 | .27 |
| 11 | TTC12 | rs2282511 | 113244177 | 174 | .32 | T | G | 0.30 | .01 | 1.65 | .10 |
| 11 | ANKK1 | rs877138 | 113256508 | 175 | .31 | C | T | 0.29 | .01 | 1.55 | .12 |
| 11 | ANKK1 | rs4938012 | 113259654 | 175 | .31 | T | C | 0.29 | .01 | 1.61 | .11 |
| 11 | ANKK1 | rs17115439 | 113264272 | 174 | .33 | A | G | 0.17 | .01 | 1.33 | .19 |
| 11 | ANKK1 | rs4938013 | 113264470 | 174 | .31 | A | C | 0.29 | .01 | 1.75 | .08 |
| 11 | ANKK1 | rs4938015 | 113264644 | 173 | .33 | T | C | 0.17 | .01 | 1.40 | .16 |
| 11 | ANKK1 | rs1800497 | 113270828 | 175 | .2 | T | C | 0.81 | .00 | .04 | .97 |
| 11 | DRD2 | rs6279 | 113281073 | 164 | .35 | G | C | 0.30 | .01 | .70 | .48 |
| 11 | DRD2 | rs1076560 | 113283688 | 175 | .14 | A | C | 0.75 | .00 | .06 | .95 |
| 11 | DRD2 | rs2283265 | 113285536 | 173 | .13 | T | G | 0.51 | .00 | .30 | .77 |
| 11 | DRD2 | rs2440390 | 113286878 | 174 | .12 | A | G | 0.47 | .03 | 1.90 | .06 |
| 11 | DRD2 | rs2075654 | 113289066 | 175 | .14 | A | G | 0.54 | .00 | .14 | .89 |
| 11 | DRD2 | rs1800498 | 113291588 | 174 | .44 | G | A | 0.44 | .01 | .71 | .48 |
| 11 | DRD2 | rs2587548 | 113292212 | 174 | .43 | G | C | 0.35 | .01 | .66 | .51 |
| 11 | DRD2 | rs1076563 | 113295909 | 175 | .43 | T | G | 0.36 | .01 | .69 | .49 |
| 11 | DRD2 | rs1076562 | 113296008 | 174 | .29 | A | G | 0.58 | .01 | .82 | .41 |
| 11 | DRD2 | rs1079597 | 113296286 | 169 | .14 | A | G | 1.00 | .00 | .00 | 1.00 |
| 11 | DRD2 | rs1079596 | 113296619 | 175 | .14 | T | C | 0.75 | .00 | -.03 | .97 |
| 11 | DRD2 | rs1125394 | 113297185 | 175 | .14 | G | A | 0.75 | .00 | -.03 | .97 |
| 11 | DRD2 | rs2471857 | 113298339 | 175 | .14 | A | G | 0.36 | .00 | -.16 | .87 |
| 11 | DRD2 | rs7103679 | 113303674 | 170 | .14 | T | C | 0.53 | .00 | .16 | .87 |
| 11 | DRD2 | rs4586205 | 113307129 | 174 | .3 | G | T | 0.86 | .01 | .56 | .57 |
| 11 | DRD2 | rs4648318 | 113313389 | 175 | .28 | C | T | 1.00 | .01 | .99 | .32 |
| 11 | DRD2 | rs11214608 | 113315355 | 174 | .45 | G | A | 0.45 | .01 | .92 | .36 |
| 11 | DRD2 | rs4274224 | 113319452 | 174 | .42 | G | A | 0.64 | .00 | -.37 | .71 |
| 11 | DRD2 | rs4581480 | 113324474 | 175 | .12 | C | T | 0.47 | .00 | -.04 | .97 |
| 11 | DRD2 | rs4648317 | 113331532 | 175 | .18 | T | C | 0.45 | .00 | .04 | .97 |
| 11 | DRD2 | rs4350392 | 113335717 | 174 | .43 | T | G | 4.60 x 10^-13^ | .00 | -.23 | .82 |
| 11 | DRD2 | rs4245149 | 113338357 | 175 | .18 | A | G | 0.45 | .00 | .04 | .97 |
| 11 | DRD2 | rs4938019 | 113341391 | 175 | .18 | G | A | 0.61 | .00 | -.07 | .94 |
| 11 | DRD2 | rs10891556 | 113352761 | 168 | .21 | A | C | 1.00 | .00 | .18 | .85 |
| 11 | DRD2 | rs6589377 | 113355736 | 175 | .3 | C | T | 0.37 | .00 | -.55 | .58 |
| 11 | DRD2 | rs4482060 | 113358211 | 170 | .37 | A | T | 0.51 | .00 | -.07 | .94 |
| 11 | DRD2 | rs4245150 | 113364647 | 175 | .3 | G | T | 0.37 | .00 | -.55 | .58 |
| 11 | DRD2 | rs4938023 | 113374847 | 175 | .29 | A | C | 0.36 | .00 | -.56 | .57 |
| 11 | DRD2 | rs12361003 | 113380818 | 175 | .35 | T | G | 0.62 | .00 | -.23 | .82 |
| 11 | DRD2 | rs2514218 | 113392994 | 175 | .27 | T | C | 0.85 | .00 | -.37 | .71 |
| 11 | DRD2 | rs2511515 | 113397655 | 175 | .31 | C | A | 1.00 | .00 | .37 | .71 |
| 22 | COMT | rs737866 | 19930109 | 175 | .25 | G | A | 0.07 | .01 | 1.61 | .11 |
| 22 | COMT | rs933271 | 19931407 | 174 | .25 | G | A | 0.31 | .00 | .45 | .65 |
| 22 | COMT | rs174675 | 19934051 | 174 | .25 | T | C | 0.31 | .00 | .51 | .61 |
| 22 | COMT | rs5993883 | 19937638 | 175 | .47 | C | A | 0.23 | .01 | 1.82 | .07 |
| 22 | COMT | rs740603 | 19945177 | 169 | .45 | C | T | 0.88 | .01 | 1.44 | .15 |
| 22 | COMT | rs165656 | 19948863 | 133 | .39 | G | C | 0.72 | .01 | 1.27 | .20 |
| 22 | COMT | rs6269 | 19949952 | 165 | .1 | C | T | 4.98 x 10^-15^ | .02 | 2.02 | .05 |
| 22 | COMT | rs2239393 | 19950428 | 175 | .35 | C | T | 1.00 | .01 | 1.15 | .25 |
| 22 | COMT | rs4680 | 19951271 | 175 | .42 | G | A | 0.88 | .01 | .79 | .43 |
| 22 | COMT | rs4646316 | 19952132 | 175 | .26 | T | C | 0.33 | .01 | 1.40 | .16 |
| 22 | COMT | rs174696 | 19953176 | 174 | .24 | C | T | 0.84 | -.02 | -1.76 | .08 |
| 22 | COMT | rs9332377 | 19955692 | 173 | .12 | T | C | 0.28 | -.02 | -1.73 | .09 |
| 23 | MAOA | rs5906729 | 43520371 | 175 | .28 | C | G | 1.00 | .01 | 1.78 | .08 |
| 23 | MAOA | rs1465108 | 43538209 | 175 | .29 | T | C | 1.00 | .01 | 1.91 | .06 |
| 23 | MAOA | rs5906957 | 43547310 | 175 | .22 | T | C | 0.63 | .01 | .78 | .44 |
| 23 | MAOA | rs909525 | 43553202 | 173 | .29 | G | A | 1.00 | .01 | 1.71 | .09 |
| 23 | MAOA | rs2235185 | 43595743 | 175 | .26 | T | C | 1.00 | .01 | 1.71 | .09 |
| 23 | MAOA | rs2072744 | 43599436 | 175 | .32 | A | G | 1.00 | .01 | 1.43 | .15 |
| 23 | MAOA | rs979605 | 43601363 | 174 | .28 | A | G | 1.00 | .01 | 1.32 | .19 |
| 23 | MAOA | rs2239448 | 43602679 | 175 | .27 | A | G | 1.00 | .01 | 1.74 | .08 |
| 23 | MAOB | rs1799836 | 43627999 | 175 | .4 | C | T | 0.02 | .00 | -.64 | .52 |
| 23 | MAOB | rs10521432 | 43633740 | 175 | .23 | T | C | 1.00 | -.01 | -1.70 | .09 |
| 23 | MAOB | rs6651806 | 43688964 | 175 | .23 | G | T | 1.00 | -.01 | -1.58 | .12 |
| 23 | MAOB | rs5905512 | 43726394 | 174 | .47 | C | T | 1.00 | -.01 | -1.14 | .26 |

*Note.* Chr = Chromosome, BP = base pair, MAF = Minor allele frequency, Min = Minor allele, Maj = Major allele.
